# Optimizing solar irradiance forecasting: ANN models enhanced with ADAM and Cuckoo search algorithm

**DOI:** 10.1371/journal.pone.0335342

**Published:** 2025-12-05

**Authors:** Muhammad Sadiq, Muhammad Jibreel Sammar, Muhammad Anwaar Saeed, Lu Chen, Junwei Liang, Mohammad Y. AlZahrani, Syed Muhammad Mohsin

**Affiliations:** 1 Shenzhen University of Information Technology, Shenzhen, China; 2 Department of Computer Science, Virtual University of Pakistan, Lahore, Pakistan; 3 School of Electronic Information, Xijing University, Xi’an, China; 4 Future Energy Technologies Institute, KACST, Riyadh, Kingdom of Saudi Arabia; 5 Department of Computer Science, University of Wah, Wah Cantt, Pakistan; 6 Standard College of Modern Sciences, Virtual University of Pakistan, Lahore, Pakistan; Khalifa University, UNITED ARAB EMIRATES

## Abstract

Renewable energy sources (RES) are being used and integrated into the electrical grid as a result of the environment’s effects and the ever-increasing demand for energy. Reliable and accurate forecasts are necessary to address environmental concerns and improve grid management due to the intermittent availability of renewable energy sources. This study focuses on improving ANN-based techniques for precise solar irradiance prediction as the prediction accuracy of an artificial neural network (ANN) is impacted by the random assignment of weights to its edges. As a result, we proposed hybrid solar irradiance forecasting models in which the cuckoo search algorithm (CSA) and adaptive moment estimation (ADAM) are used to optimize the weights assigned to the ANN’s edges. Two models were presented in this study namely: ADAM-optimized ANN model and a novel two-stage optimization technique known as CSA-ADAM optimized ANN model for accurate and reliable forecasting of solar irradiance. Both models were tested using actual weather data, and standard error metrics like mean squared error (MSE), mean absolute percentage error (MAPE), mean absolute error (MAE), and root mean square error (RMSE) to assess their accuracy. The outcomes demonstrate that ADAM-optimized ANN model produced MSE = 0.52, MAPE = 0.18%, MAE = 0.64, and RMSE = 0.72, and CSA-ADAM optimized ANN model obtained MSE = 0.25, MAPE = 0.17%, MAE = 0.43, and RMSE = 0.50. We evaluated the practicality of both models by comparing their average prediction times using the same test dataset. While the ADAM-optimized ANN model took an average of 0.1093 ± 0.0085 seconds to make predictions on the test data, the CSA-ADAM optimized ANN model took 0.1110 ± 0.0058 seconds. These findings demonstrate that using CSA to optimize the ANN weights increases the accuracy of solar irradiance predictions.

## 1 Introduction

Conventional energy sources that rely on fossil fuels are ecologically unsustainable and negatively affect the environment [1]. This has led to a recent surge in research on alternative energy sources. In addition, the global energy crisis is getting worse since technological and economic advancements heavily rely on energy availability, which is necessary for global urbanization and industrialization. On the other hand, there is a global energy deficit as a result of population growth [2]. As the demand for energy rises, so does the use of fossil fuels. Brown energy is produced using fossil fuels, such as coal, natural gas, and oil, which raises costs and damages the environment. One abundant and reasonably priced source of renewable energy is solar power. Compared to brown energy, green energy has a smaller carbon footprint [3,4]. Given the long-term effects of carbon emissions, nations have imposed high taxes on carbon emissions from conventional power plants [5–7]. Developing strategies to lower energy costs and guarantee environmental sustainability presents major challenges for researchers and policymakers.

The research community has been working on amazing green energy solutions [8–10]. According to current research trends, combining renewable energy sources with conventional fossil fuel-based energy sources is the most efficient and environmentally friendly way to meet human energy needs [11–13]. The most common renewable energy source that can be used is solar energy. However, the production of solar energy is significantly impacted by erratic weather conditions. Because solar radiation varies and is influenced by weather patterns in various time zones, solar energy production is sporadic [14,15]. Despite its apparent low cost, solar energy’s intermittent nature presents a major barrier to its integration into the current energy system [16]. Therefore, to minimize carbon emissions, lower grid operating costs, reduce the use of electricity reserves through improved generation planning, ensure the electricity grid operates reliably and securely, and minimize the gap between electricity supply and demand, accurate forecasting of renewable energy generation is essential [17–21].

Cost-effective power generation is crucial to a nation’s development [22,23], and solar energy can generate power without emitting carbon dioxide [24,25]. Numerous artificial intelligence (AI)-based techniques, such as neural networks (NN), support vector regression (SVR), convolutional neural networks (CNN), gated recurrent units (GRU), long short-term memory (LSTM), and multi-layer perceptrons (MLP), have been proposed by the research community for the prediction of solar irradiance [26]. Hourly irradiance has been predicted more recently using hybrid approaches like deep neural networks (DNN) and extreme gradient boosting trees (XGBT) [27].

In this study, we have presented two solar irradiance forecasting models namely; ADAM-optimized ANN model and novel two-stage optimization technique known as CSA-ADAM optimized ANN model. First model is a baseline model whereas second one is our proposed model. The selection of CSA and ADAM was based on their complementary and distinct contributions to neural network optimization. ADAM is a popular gradient-based optimizer that uses momentum and adjusts learning rates for every parameter to help models converge effectively. It is not without drawbacks, though; if the network’s initial weights aren’t configured correctly, ADAM may have trouble and occasionally result in unstable training or become stuck in less-than-ideal solutions, particularly in complex scenarios. CSA can help with that. Inspired by nature, CSA explores the solution space more widely and haphazardly by combining cuckoo-like behavior with Lévy flights.

Because of this, it is excellent for preventing early convergence and identifying more suitable training starting points. The model gains from a clever combination of local fine-tuning and global exploration by initializing weights with CSA and then fine-tuning them with ADAM. In addition to enhancing training stability and convergence, this hybrid approach increases the model’s capacity for generalization, which is especially advantageous for tasks like solar irradiance prediction. Main contributions of this study are summarized below.

- We provide a comprehensive overview of the current literature on solar energy forecasting.
- The literature study shows that artificial neural networks experience a decrease in prediction accuracy when processing highly intermittent solar radiation data.
- We proposed a reliable solar irradiance prediction algorithm called CSA-ADAM optimized ANN model, in which the weights of the ANN edges are optimized using CSA and ADAM optimizer.

The remainder of the article is structured as follows. Sect 2 describes the existing literature about solar irradiance prediction. Sect 3 defines the motivation and problem statement, while Sect 4 provides a comprehensive overview of an artificial neural network, cuckoo search algorithm (CSA), dataset characterization, and our proposed system model. Sect 5 explains the simulation setup and methodology for this study, whereas Sect 6 presents the simulation results and associated discussion. The study concludes in Sect 7, accompanied with future research directions.

## 2 Literature review

The study at [28] examines the difficulties arising from the fluctuations in solar radiation that affect photovoltaic installations and emphasizes the need for accurate forecasting to improve grid management. The research achieves significant ramp forecast skill scores by constructing a forecasting model that utilizes LSTM networks for different weather situations. Comparisons with conventional forecasting approaches show that on-site measurement devices are essential for accurate predictions, while the effectiveness of features can vary with weather conditions. The study recognizes imbalances in the data sets resulting from certain regional weather patterns and proposes resampling methods to improve the efficiency of the model. This work presents innovative approaches for the integration of solar energy into power systems through the fusion of all-sky images and numerical data in machine learning applications.

The authors of [29] present a method using artificial neural networks (ANN) to improve operational predictions of direct normal irradiance and address the shortcomings often found in numerical weather prediction models [30]. The models created integrate meteorological and aerosol data from ECMWF and CAMS and produce 10-minute DNI forecasts that show a significant improvement in accuracy compared to the original down scaled forecasts. The assessments carried out at several locations in southern Portugal confirm the effectiveness of the model and achieve significant metrics such as R^2^, MAE and RMSE. This approach enables trustworthy predictions, allowing solar energy producers and grid operators to make more informed decisions about energy production and management.

In [31], artificial neural networks and a hybrid ANN-based genetic algorithm (ANN-GA) are used to predict the global horizontal irradiance (GHI) in Albion, Mauritius, at 15-minute intervals using ground-based measurement data. The ANN model showed superior performance on several statistical parameters, with remarkable correlation coefficients and minimal error rates compared to the hybrid strategy. Validation with satellite data at a different location underpinned the efficiency of the ANN model and emphasized its robustness in predicting solar irradiance. The results emphasize the importance of accurate forecasting for improving energy management and integrating renewable energy sources into the power grid.

The increasing global demand for energy, fueled by population growth and economic development, necessitates a transition to renewable energy sources such as photovoltaics (PV). The integration of photovoltaic systems into the electricity grid offers environmental and economic benefits. However, their intermittent nature can jeopardize system stability at high supply levels. study at [32] presents an innovative forecasting model for ultra-short-term GHI that integrates the GRU and temporal convolutional network (TCN) architectures. The study compares univariate and multivariate GRU-TCN models with alternative deep learning techniques and shows that the univariate model, which relies solely on historical GHI data, achieves a lower MAE of 23.02 W/m^2^, compared to the 25.67 W/m^2^ of the superior multivariate model. The results show that the univariate GRU-TCN method is suitable for reliable one-step prediction and improves the accuracy of GHI predictions. Future studies could investigate hybrid models, incorporate additional meteorological variables and focus on multi-step predictions to improve the reliability of clean energy sources.

In [33], the authors employ a full year of GOES-R satellite data to produce 5-minute cloud cover statistics for seven surface radiation budget network sites, focusing on GHI for now casting and short-term forecasting. Two convolutional neural networks (CNN) were evaluated: one with the spectral cloud optical property estimation method and the other without this extension. The SCOPE model showed an average RMSE of 44.7 W/m^2^, while the standard model reached 68.9 W/m^2^. The results demonstrate the effectiveness of CNNs in analyzing satellite data to estimate local ground irradiance, with the SCOPE-enhanced model significantly outperforming models using only longwave radiation channels. Subsequent breakthroughs led to the creation of a hybrid SCOPE-CNN-ConvLSTM model, which showed a significant improvement in prediction accuracy, especially under partly cloudy skies, with prediction capabilities between 38% and 42%.

A fuzzy logic classifier is presented in [34] that is used to classify daily irradiance patterns, mimicking human decision making. The classifier emphasizes language interpretability and accuracy and uses data mining and supervised learning methods to develop an initial system. Simplification methods, such as fuzzy classifiers with insufficient rule base, lead to a concise final model that competently resolves the ambiguity of daily irradiance data. The classifier divides the days into four main categories cloudy, partly cloudy, partly sunny and sunny—each of which is subdivided into the subcategories Low, Medium and High, resulting in a total of 12 rules. Following the categorization, a neuro-fuzzy algorithm predicts the performance ratio PR of photovoltaic systems depending on the time of day, the maximum ambient temperature and the period of use. This model has a commendable prediction accuracy and effectively detects abnormal behavior of the system during the summer of 2019, using seven years of training data and two years of test data.

The growing importance of renewable energy sources, especially solar energy, is driven by global climate change and the need to reduce greenhouse gas emissions. In [35], an improved deep learning model for solar irradiance prediction is presented that utilizes convolutional properties and results in a reduction of the MAE by 8.32 compared to conventional models. A thorough investigation of current mathematical models and data emphasizes the ability of deep learning techniques to improve prediction accuracy, which is essential for efficient renewable energy management. The work focuses on the importance of data quality and suggests further research opportunities, such as investigating hybrid models and incorporating additional meteorological variables to improve scalability and applicability in different geographical areas.

The requirements for accurate energy forecasting in renewable energy generation, focusing on very short-term estimates of solar irradiance, about ten minutes in advance is presented in [36]. To improve the prediction accuracy, two ANN models have been created: the CNN and the LSTM. With a RMSE of 52.58 W/m^2^ during the test i.e., an improvement of 8.16% over a comparative model for persistence indicates that the CNN performs better than the LSTM model. On 59% of the days tested, the mean error of the CNN model was below 2%, with a significant proportion of these days having a mean error of 2.75. All in all, the results suggest that this forecasting tool can successfully reduce uncertainty in photovoltaic power generation, allowing for better integration into the power grid and better energy management in general.

Meteorological data from five cities in Bangladesh is utilized in [37] to develop a neural network-based solar radiation prediction system. Three models GRU, LSTM and RNN were trained and their performances were evaluated using different criteria. With a MAPE value of 19.28%, the GRU model performed better than the others. The study emphasizes how better predictions of solar radiation could boost energy production and how more and diverse data sets could help future research to increase model accuracy.

Precise forecasting of solar energy is crucial for efficient power grid management and maintaining a constant supply to consumers [38]. Conventional statistical and machine learning techniques often prove insufficient due to the unpredictable nature of solar data, necessitating an investigation of deep learning architectures such as LSTM, GRU, RNN, CNN and GAN to improve prediction performance. This study shows that deep learning methods outperform traditional methods, especially when integrated with methods to improve feature extraction and data augmentation. Critical determinants of model accuracy are prediction horizon, weather classification and input feature optimization, while hybrid models show improved performance by integrating both temporal and spatial data attributes. The results show that while deep learning algorithms have the potential to address time series prediction problems, further research is needed to improve their application in solar energy systems.

Prediction accuracy of photovoltaic solar power generation is analyzed in [39] using ANN and LSTM models, focusing on their performance over three short-term forecast horizons: 1, 15 and 60 minutes. The results show that the two models have slightly different accuracy. The LSTM generally performs better, especially at shorter forecast horizons, and has a MAPE of 19.5%. The study also shows that the amount of exogenous meteorological input variables has no significant influence on the forecast accuracy, which means that simpler models can be successful even without much sensor data. Even though both models show decreasing accuracy at longer forecast horizons, the study emphasizes the need for more complicated LSTM structures and larger training data sets to correctly identify long-term trends. All in all, the results provide an insightful analysis for next solar power forecasting studies and point to possible directions for optimizing parameters and improving model performance through hybrid methods.

As non-renewable energy sources are near to an end, the world’s attention is turning to renewable alternatives, particularly solar energy, which is expected to be central to the future energy supply [40]. Integrating solar energy into the power grid is problematic due to its inherent unreliability, making microgrid systems a more efficient approach to managing the complexity. Accurate forecasting models for solar energy are critical to improving the reliability of solar installations in these microgrids, as forecasting uncertainty remains a major challenge. This paper evaluates more than 100 solar irradiance and power prediction models and classifies them based on their characteristics, performance indicators, and respective advantages and disadvantages to help researchers select appropriate models for specific locations and time periods. It emphasizes the effectiveness of ensemble and hybrid models, which offer improved predictive capabilities compared to conventional methods. Various artificial intelligence tools are used to analyze large data sets and increase the accuracy of solar energy forecasting.

The study at [41] presents an innovative method for short-term solar irradiance prediction that integrates bayesian optimized attention-dilated LSTM with Savitzky-Golay filtering and uses data from a solar irradiance sensor in Douala, Cameroon. The method improves the quality of the raw data by incorporating solar irradiance factors and using multiple deep learning models, and finally shows that the hybrid model with attention mechanisms and dilated convolutional layers achieves higher accuracy. The proposed model outperforms previous research with a symmetric mean absolute percentage error of 0.6564 and a RMSE of 22.9445, which is a significant improvement in prediction accuracy. The research improves the field by presenting efficient data pre-processing techniques and a benchmark dataset to help both researchers and solar plant management refine forecasting capabilities. Future endeavors aim to extend the applicability of the model to more sites and investigate the integration of real-time data for improved flexibility.

Photovoltaic panels are an efficient technology for generating renewable energy by converting solar radiation into electricity, although their efficiency depends on variables such as the quality of the panels and the local solar radiation [42,43]. Accurate prediction of solar radiation is crucial for improving the design and management of solar power plants. This study investigates solar irradiance prediction as a machine learning challenge and uses a three-year dataset from Izmir, Turkey to evaluate several deep learning and conventional machine learning methods. The comparative study shows that MLP model performs best in predicting GHI with a RMSE of 0.78, followed by the random forest regressor and the CatBoost gradient boosting approach. The results show that the proposed MLP model can accurately predict the GHI values, which facilitates informed decision making for the installation of solar modules.

## 3 Motivation and the problem statement

### 3.1 Motivation

A precise and reliable forecast is essential for an accurate prediction of solar radiation, as solar irradiance and climatic parameters fluctuate considerably. Research has presented various models for predicting solar irradiance using artificial neural networks. The literature review reveals that ANN based solar irradiance forecasting models experience a decrease in prediction accuracy when confronted with highly fluctuating solar irradiance data, and that the random assignment of initial weights to the edges of the ANN further affects the prediction performance of the model. Therefore, this study proposes a novel two-stage optimization technique to assign CSA and ADAM optimized weights to the edges of the ANN to improve its prediction accuracy and reliability.

CSA and ADAM were chosen for their fundamentally different but complementary optimization behaviors. ADAM is a popular gradient-based optimizer that allows neural networks to converge effectively and reasonably quickly by incorporating momentum and adjusting learning rates for each parameter. Nevertheless, ADAM’s reliance on the initial weight configuration is one of its recognized drawbacks. ADAM may show unstable training behavior or converge to suboptimal local minima if weights are not initialized properly, especially in complex, non-convex landscapes like those encountered in ANN training.

On the other hand, CSA is a nature-inspired metaheuristic that combines Lévy flight-based random walks with the brood parasitism behavior of cuckoos. This makes CSA very effective for global exploration and preventing premature convergence by allowing it to conduct a wide and random search of the solution space. CSA is a good option for initializing ANN weights in a way that enhances the starting point for additional fine-tuning because of its capacity to break free from local optima and more fully explore the search space. A more robust optimization result depends on the diversity of candidate solutions, which is encouraged by its stochastic nature and selection mechanisms (such as elitism and nest abandonment).

Our proposed CSA-ADAM optimized ANN model successfully combines the advantages of both global and local search strategies by first using CSA to identify a high-quality set of initial weights, and then using ADAM’s gradient-based updates to refine those weights. The well-known optimization principle of striking a balance between exploration and exploitation is the foundation of this two-stage method. In terms of algorithms, this results in enhanced generalization, decreased training errors, and better convergence behavior.

### 3.2 Problem statement

The aim of this study is to create, implement and validate a solar irradiance forecasting model that exploits ADAM and CSA for the optimal weights assignment to the edges of artificial neural network in order to obtain a reliable and accurate solar irradiance forecasting model.

## 4 Proposed system model

### 4.1 Artificial neural network (ANN)

**Fig 1:**
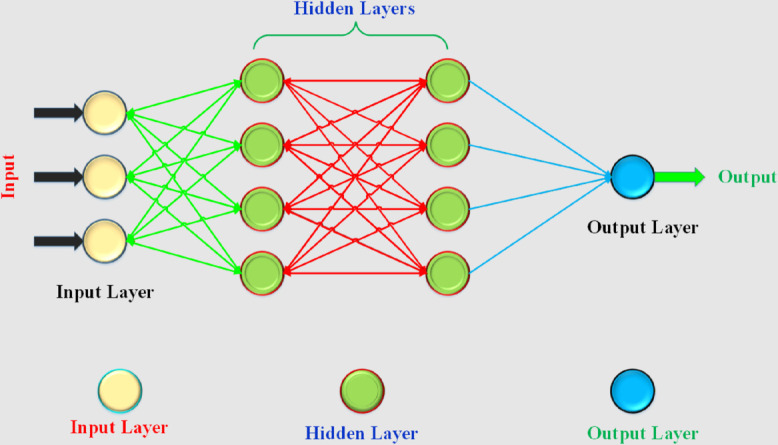
ANN with 2 hidden layers.

ANN consists of interconnected nodes, known as artificial neurons, which mimic the functioning of the human nervous system. The nodes of the artificial neural network have a complicated connection structure via edges that serve as lines for transmitting signals to the successive artificial neurons. The neural network then processes these signals logically. This information can be passed on to the subsequent cell for further processing or for final output. Artificial neural networks can be constructed with an input layer, two hidden layers with four neurons each and an output layer that can accommodate one or more nodes, as shown in Fig 1. The most important factors of an ANN are the number of hidden layers, number of iterations, and the learning rate. Various activation functions are crucial for the complex functioning of artificial neural networks (ANNs). The best-known activation functions include softmax, the sigmoidal stochastic linear error unit, the exponential linear unit and Ripple. Artificial neural networks can encounter the following problems if the weight value *w*_*i*_ is chosen randomly.

- The poor precision as a result of unreliable inputs in prediction process.
- Misallocation of initial weights between a network’s layers can have a negative effect on its performance.

### 4.2 Cuckoo search algorithm (CSA)

**Fig 2:**
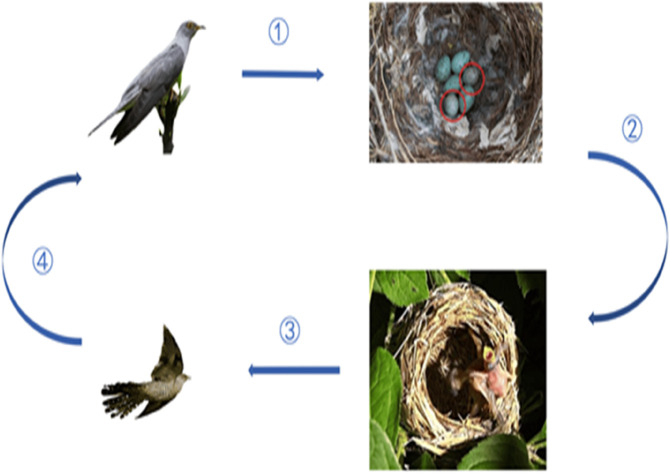
Cuckoo bird hatched cycle.

**Fig 3:**
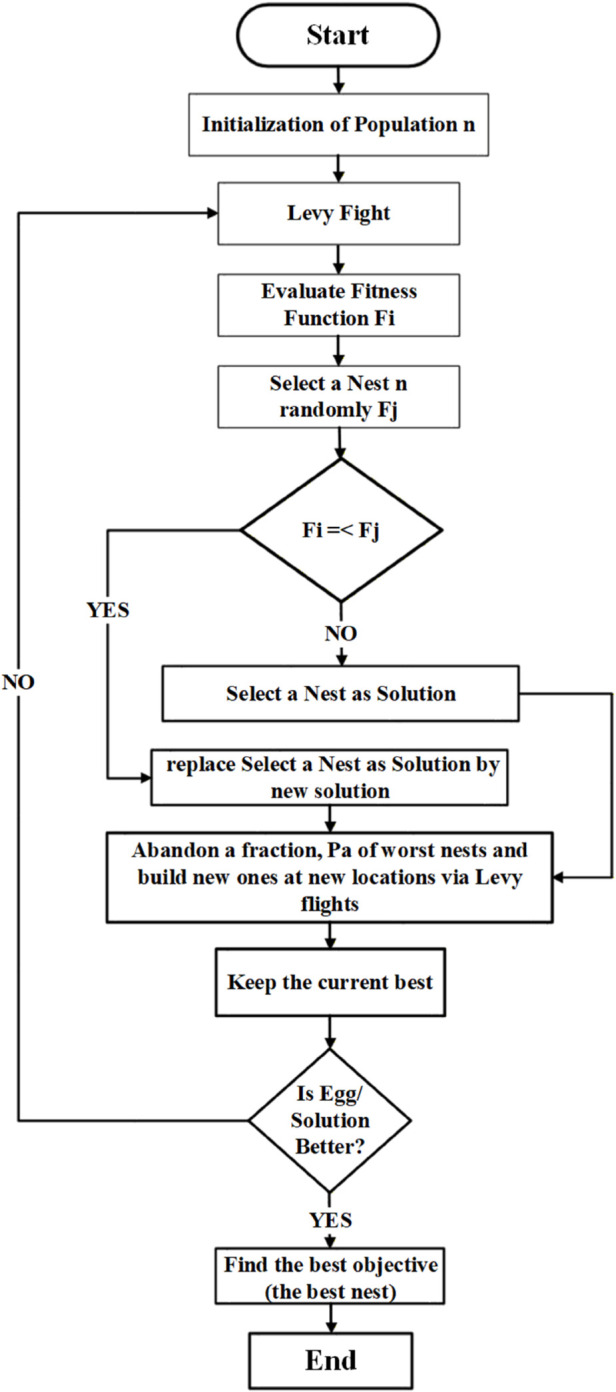
Flow diagram of cuckoo search algorithm.

The cuckoo search algorithm (CSA) is a meta-heuristic algorithm [44]. Certain parasitic cuckoo females have the ability to mimic the egg pattern and coloring of their host birds. The process steps of the CSA are shown in Fig 2. Within this algorithmic structure, the following two terms are considered: 1. cuckoo bird 2. host bird. Flow diagram of cuckoo search algorithm is shown in Fig 3.

Cuckoo birds and host birds (other bird species) are two species. Cuckoo birds are known to use the nests of other bird species to hatch their offspring. This increases both their lifespan and their productivity. When the host bird discovers the cuckoo eggs:

- The egg might be dropped by the host bird.
- Leave the nest and develop a new-one.

### 4.3 Dataset pre-processing

Important pre-processing procedures were performed on the dataset prior to training in order to guarantee data quality and enhance model performance. Interpolation and outlier removal were used to deal with missing and inconsistent values. To help stabilize and expedite the ANN’s training process, all input features were normalized using min-max scaling to maintain values between 0 and 1. To match the solar irradiance forecasting time-frame, the data was arranged into 10-minute intervals per day for one week.

Along with time-related features, we selected pertinent meteorological variables for feature selection, including temperature at different elevations, earth skin temperature, and PRECTOTCORR. By examining correlation coefficients between features and eliminating or combining those that showed a strong correlation, we were able to address multicollinearity. This straightforward but efficient method reduced redundancy and guaranteed that the final set of input features was informative, which improved the prediction accuracy and generalization of the ANN models.

The original dataset contained 1431 values for various weather characteristics collected from 01 April 2018 to 01 March 2022. This data was distributed so that each value represented the weather at a specific time of day and was recorded at 10-minute intervals per day for one week. Insights into the data of the solar irradiance dataset downloaded from [45] for the period from 01 April 2018 to 01 March 2022 can be found in Table 1 showing the following variables.

- *T*2*M* (temperature at 2 meters height)
- *T*2*M*_*MAX*_ (maximum temperature at 2 meters height)
- *T*2*M*_*MIN*_ (minimum temperature at 2 meters height)
- TS (surface temperature)
- PRECTOTCORR (corrected precipitation sum)

**Table 1:** Data insights.

Parameter	T2M (^∘^C)	${T2M}_{MAX}$ (^∘^C)	${T2M}_{MIN}$ (^∘^C)	TS (^∘^C)	PRECTOTCORR (mm/day)
count	1431.000000	1431.000000	1431.000000	1431.000000	1431.000000
mean	9.312642	17.310175	2.441670	9.766723	1.113564
std	10.060776	10.975939	8.954626	10.841513	2.476481
min	-18.010000	-14.770000	-20.150000	-16.050000	0.000000
25%	0.805000	8.190000	-4.595000	-0.100000	0.010000
50%	8.450000	17.440000	1.150000	9.030000	0.150000
75%	19.145000	27.385000	11.035000	20.315000	1.000000
max	27.380000	36.600000	18.960000	28.760000	24.830000

#### 4.3.1 Dataset visualization.

**Fig 4:**
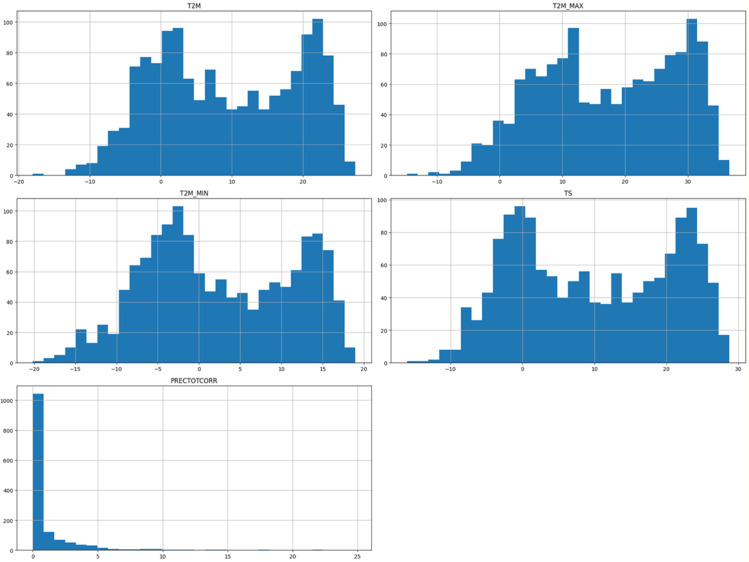
Histogram for numerical variables of solar irradiance data.

**Fig 5:**
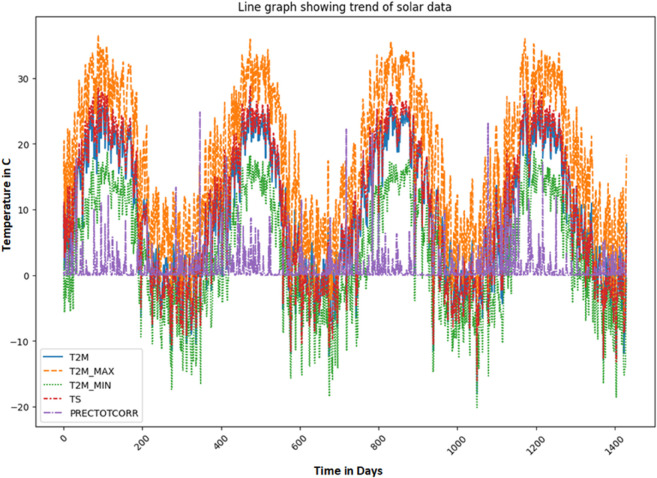
Line graph showing trend of solar irradiance data.

**Fig 6:**
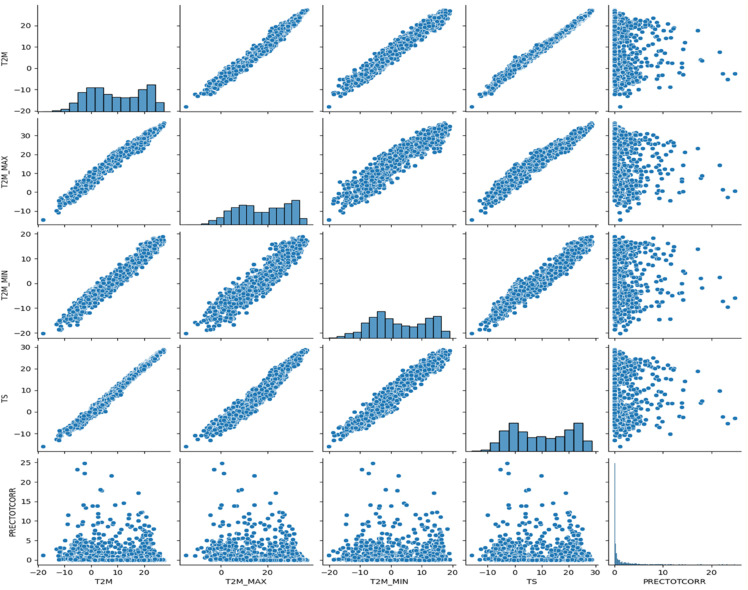
Pair plot for numerical variables of solar irradiance data.

**Fig 7:**
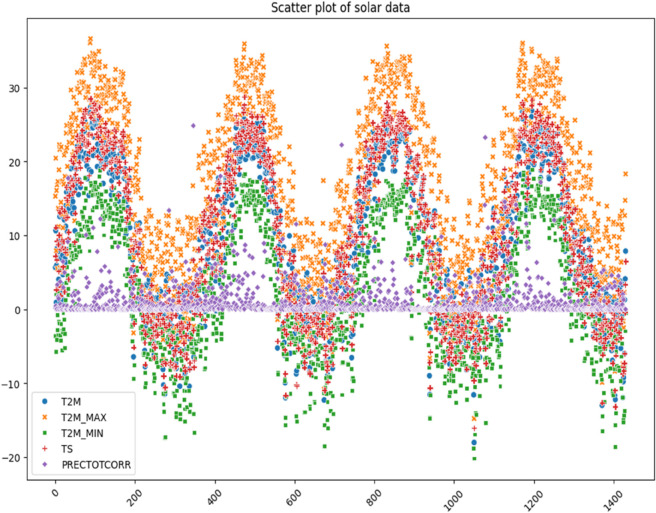
Scatter Plot of solar irradiance data.

**Fig 8:**
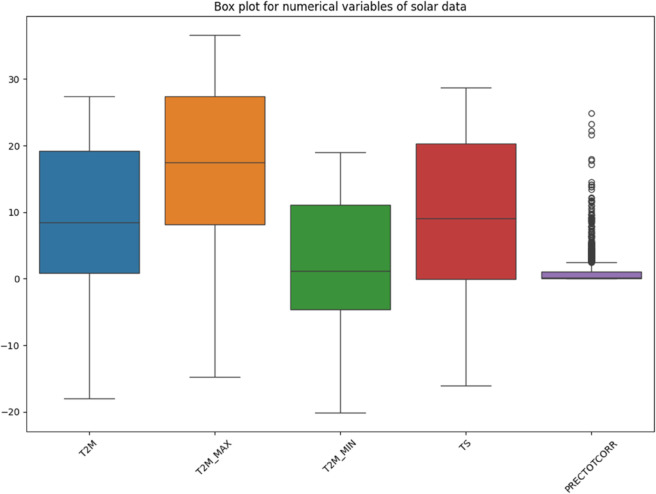
Box Plot for numerical variables solar irradiance data.

**Fig 9:**
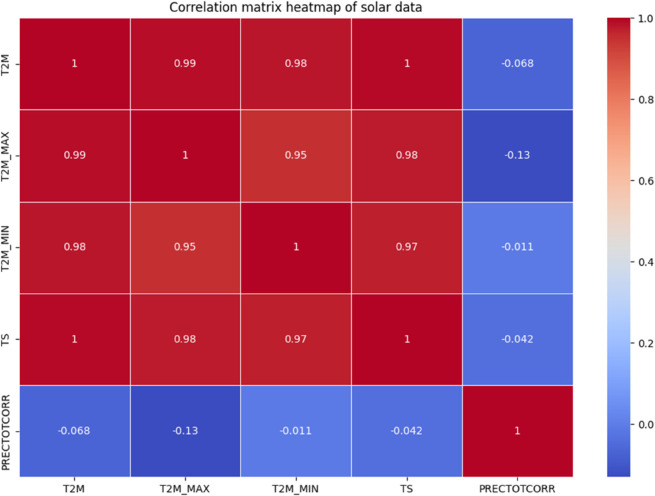
Correlation matrix Heat-Map of solar irradiance data.

The visualization of solar data is crucial to reveal inherent patterns, trends and correlations within the data set. The use of various graphical methods enables the effective interpretation of complex data, leading to better predictions and informed decisions. In this study, different visualization techniques for the analysis of solar data are explored, providing insights into the dynamic characteristics of solar irradiance and its determinants. Various prominent visualizations of the used solar data set are shown in Figs 4, 5, 6, 7, 8, and 9.

- Histograms: It provide a visual representation of the distribution of values for the different variables in the solar data.
- Line graph: The line chart shows the development of each variable over a certain period of time. Each variable is shown with a different color, indicating the fluctuations over time. This visualization helps to identify patterns, trends or cycles in the temperature and precipitation data, such as seasonal changes or anomalies in the prediction of solar radiation over the years.
- Pair plot: The pair plot is a matrix of scatter plots that show the pairwise relationships between different variables together with the distribution of each variable in the form of histograms on the diagonal. This plot is useful for recognizing correlations, patterns and potential outliers between variables.
- Scatter plot: The scatter plot visualizes the values of the variables over time. Each variable is represented by a different symbol and a different color. This type of chart is particularly useful for recognizing trends, outliers and clusters within the data for the different variables used in the solar radiation forecast.
- Box plot: The box plots provide a summary of the numerical data by their quartiles and identify outliers. Each boxplot represents a variable and shows the median, the 25th and 75th percentile, the range of the data and the outliers. This allows the dispersion and center of the data to be compared for different variables used to predict solar radiation.
- Correlation matrix heat-map: The heat map of the correlation matrix illustrates the strength and direction of the correlation between the individual pairs of variables. A values close to 0 indicate no correlation whereas, a value close to 1 or -1 indicates a strong positive or negative correlation. This heatmap enables quick identification of strongly correlated variables, which can be important for regression analysis or the identification of multicollinearity in modeling contexts.

### 4.4 Proposed system model

**Fig 10:**
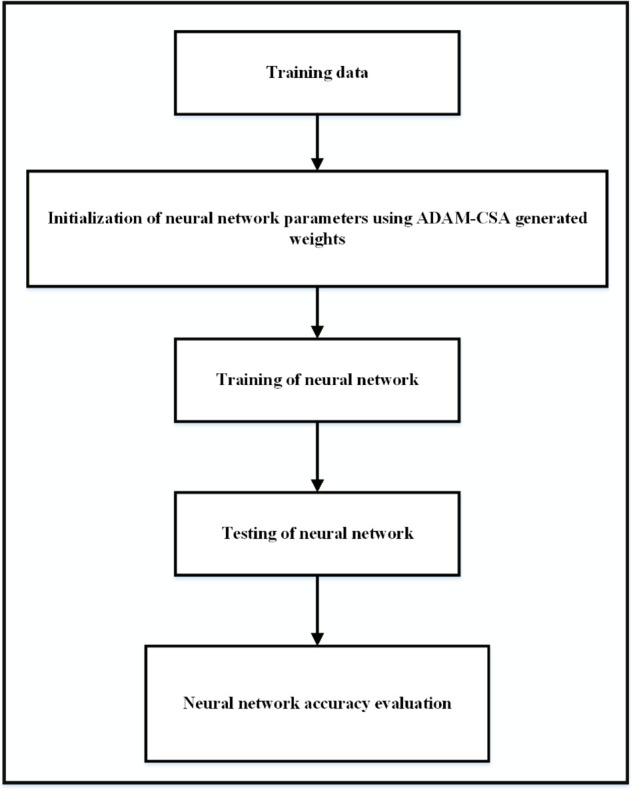
Basic block diagram of our proposed CSA-ADAM optimized ANN model.

**Fig 11:**
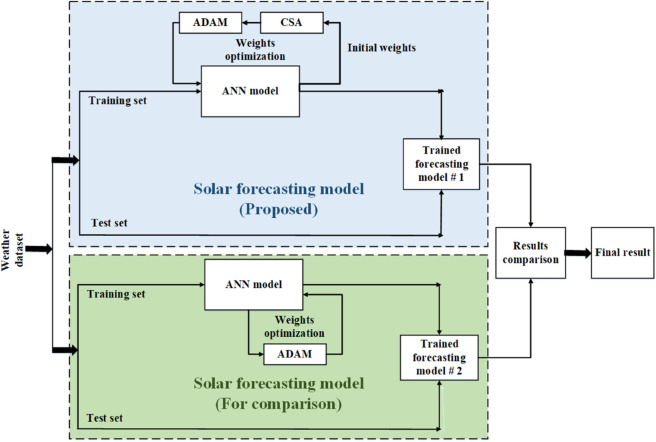
Proposed system model for solar irradiance prediction.

This study integrates the CSA and ADAM algorithms with ANN. To ensure a reliable and accurate prediction of solar irradiance, the initially randomly assigned weights of the neural network edges are refined by improved weight assignments using the CSA and ADAM algorithms. The method is used to determine the initial weights connecting the different layers of the artificial neural network. Fig 10 depicts the basic block diagram of our proposed CSA-ADAM optimized ANN model. The proposed solar irradiance prediction model, shown in Fig 11, depicts the functionalities of machine learning (ML), artificial neural networks (ANN) with a meta-heuristic algorithm known as cuckoo search algorithm (CSA) and gradient-based optimization technique ADAM. As a result, the proposed forecasting method exhibits a significant degree of precision and is expected to improve the accuracy and reliability of solar energy forecasts.

**Algorithm 1 Pseudo-code of baseline and proposed solar forecast models.**

1: **function** Main

2:   Load *weather_dataset*

3:   Split dataset into *training_set*, *test_set*

4: Proposed model - CSA-ADAM optimized ANN model i.e., model1

5:   *initial_weights* ← OptimizeWeights(CSA-ADAM)

6:   *model1* ← TrainANN(training_set, initial_weights)

7:   *forecast1* ← TestANN(model1, test_set)

8: Baseline model - ADAM-optimized ANN model i.e., model2

9:   *model2* ← TrainANN_ADAM(training_set)

10:   *forecast2* ← TestANN(model2, test_set)

11:   *result* ← CompareResults(forecast1, forecast2)

12:   Present the final comparison outcome for analysis

13: **end function**

14: **function** OptimizeWeights(CSA-ADAM)

15:   Initialize a population of candidate weights

16:   **for** each generation **do**

17:    Evaluate fitness using ANN prediction error

18:    Apply Cuckoo Search rules to generate new solutions

19:    Use ADAM to fine-tune selected weights

20:   **end for**

21:   **return** Best-performing weights

22: **end function**

23: **function** TrainANN(training_set, weights)

24:   Initialize ANN using *weights*

25:   **for** each epoch **do**

26:    Perform forward pass on *training_set*

27:    Compute loss

28:    Backpropagate error and update weights

29:   **end for**

30:   **return** trained ANN model

31: **end function**

32: **function** TrainANN_ADAM(training_set)

33:   Initialize ANN with random weights

34:   **for** each epoch **do**

35:    Forward pass and compute loss

36:    Update weights using ADAM optimization

37:   **end for**

38:   **return** trained ANN model

39: **end function**

40: **function** TestANN(model, test_set)

41:   Predict output using the trained *model*

42:   Calculate error metrics (MAPE, RMSE, MAE and MSE)

43:   **return** forecast results and performance metrics

44: **end function**

45: **runction** CompareResults(forecast1, forecast2)

46:   Compare error metrics

47:   Determine which model performs better

48:   **return** summary of comparison

49: **end function**

In this paper, a CSA-ADAM optimized ANN model for solar energy prediction is proposed, in which the initial weights at the edges of the ANN between the different layers are determined using the cuckoo search algorithm, a meta-heuristic method. Assigning appropriate weights to the edges of the artificial neural network improves the accuracy of solar energy predictions. Pseudo-code of baseline model (ADAM-optimized ANN model) and our proposed solar forecast model (CSA-ADAM optimized ANN model) is shown in the following Algorithm 1 where model1 and forecast1 are related to our proposed CSA-ADAM optimized ANN model and model2 and forecast2 are relevant to the ADAM-optimized ANN model (baseline model). forecast1 and forecast2 are compared in the end to prove the supremacy of our proposed solar forecast model. Detailed discussion and comparative analysis of the results are given in Sect 6.

## 5 Simulation setup and methodology

The data set for solar irradiance was downloaded from [45], with the period of the data sets ranging from 01 April 2018 to 01 March 2022. The entire data set was divided into 67% for training and the remaining 33% for testing purposes. The simulations are performed for the following 2 cases.

- Case 1: ANN model for the prediction of solar irradiance using a random determination of the weights at the edges of the ANN. In this case, the weights of the edges were optimized using a gradient-based optimization algorithm, the adaptive moment estimation (ADAM-optimized ANN).
- Case 2: ANN model for prediction of solar irradiance using assignment of CSA optimized weights to the edges of ANN (CSA-ADAM optimized ANN).

The ANN base model with 2 hidden layers was created, and the solar data sets from [45] were loaded. The model was thoroughly tested on a high performance system with a Core i7 processor, 12 GB RAM and an exceptionally fast clock speed of 3.5 GHz. Python is used as the programming language in this study. Model accuracy was evaluated using standard metrics such as MAPE, RMSE, MAE and MSE.

## 6 Results and discussion

**Fig 12:**
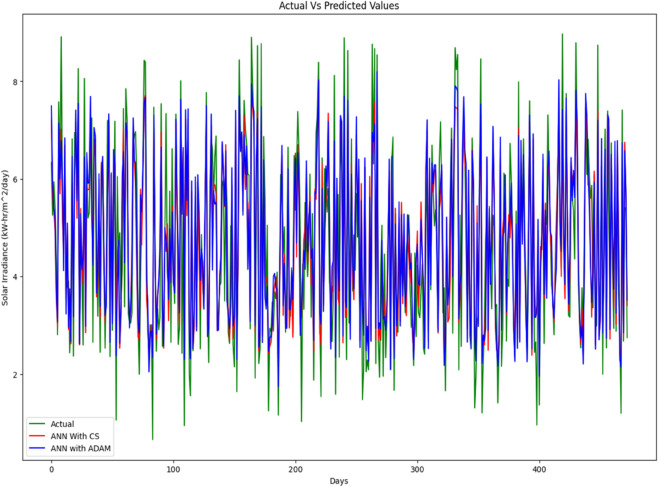
Actual vs. forecasted solar irradiance.

**Fig 13:**
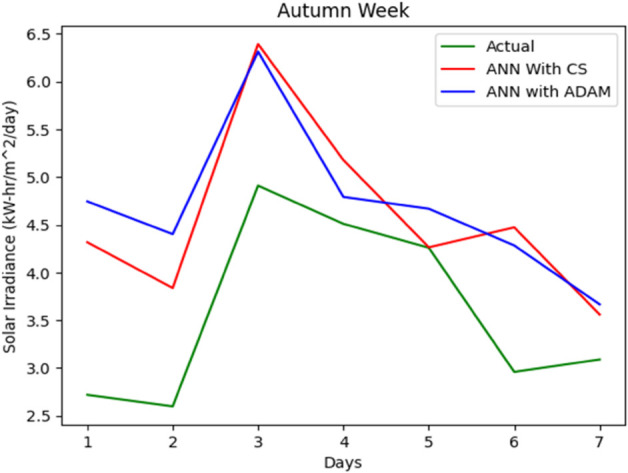
Actual vs. forecasted solar irradiance for one week of Autumn Season.

**Fig 14:**
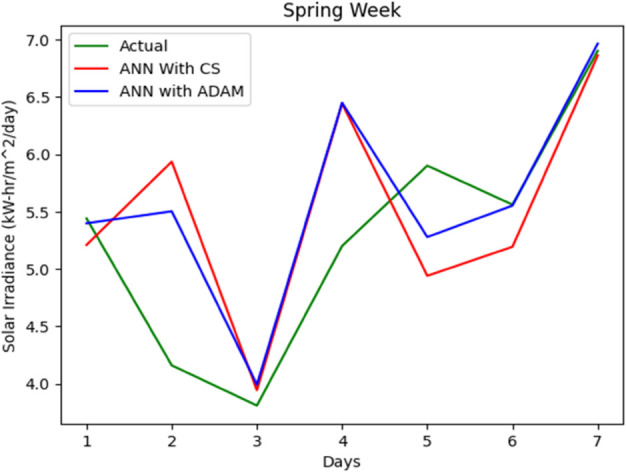
Actual vs. forecasted solar irradiance for one week of Spring Season.

**Fig 15:**
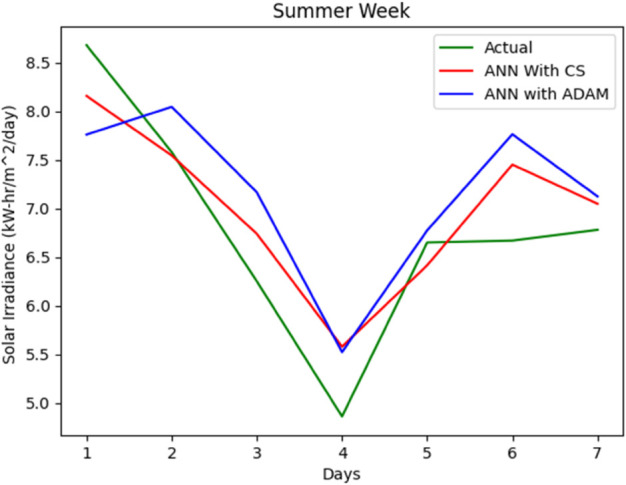
Actual vs. forecasted solar irradiance for one week of Summer Season.

**Fig 16:**
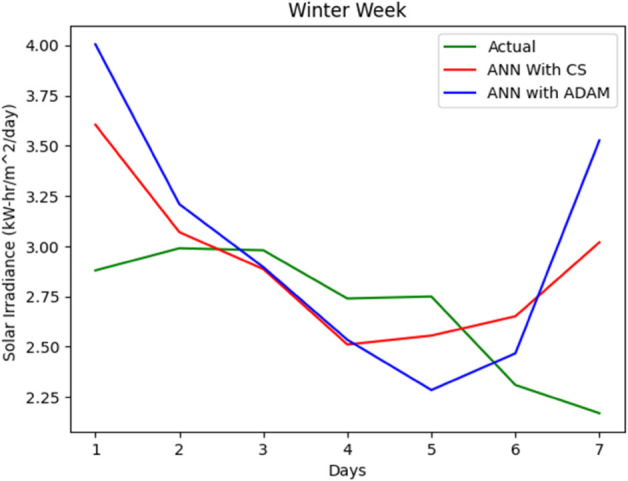
Actual vs. forecasted solar irradiance for one week of Winter Season.

The entire data set is split into 67% for training and 33% for accuracy testing. A series of simulations were performed to evaluate the accuracy of our proposed solar irradiance prediction algorithm. The same platform and test data sets were used for all experiments. An ANN model tailored to solar irradiance prediction was developed and constructed using ADAM-optimized ANN and CSA-ADAM optimized ANN technique. To ensure precision and reliability, we used well established error measurement techniques to thoroughly analyze the results obtained. The prediction of solar radiation was carried out over a period of one week and across all seasons: Autumn, Spring, Summer, and Winter. With a one-year data set having four distinct segments of spring, summer, autumn, and winter, predicting the fluctuating pattern of solar irradiance between the four seasons was a major challenge for the experiment. One-week forecast results for the autumn, spring, summer and winter seasons are shown in Figs 12, 13, 14, 15, and 16, representing actual vs. forecasted solar irradiance. It is observed that our proposed hybrid CSA-ADAM optimized ANN model performs better in terms of solar irradiance prediction accuracy.

In Figs 12, 13, 14, 15, and 16, the green lines show the actual values of solar irradiance. The blue lines, represent the results of ADAM-optimized ANN (baseline) model. Whereas, red lines show the predicted solar irradiance values using CSA-ADAM optimized solar irradiance forecasting model.

### 6.1 Forecasting accuracy comparison

The researchers in [46] created an ANN based model to predict solar irradiance. An ANN model was trained where the weights between the edges were randomized in [46]. The study [47] is based on the use of an ANN and support vector regression (SVR) to predict solar irradiance. In this study, we proposed two models: (1) ADAM-optimized ANN model (baseline): An artificial neural network whose edges are randomly weighted, and (2) a CSA-ADAM optimized ANN model (proposed): An artificial neural network whose edges are assigned CSA and ADAM optimized weights.

Table 2 shows that our proposed CSA-ADAM optimized ANN model has higher solar irradiance prediction accuracy. The results of our proposed CSA-ADAM optimized ANN model for solar irradiance prediction are MSE = 0.252231, MAPE = 0.17055%, MAE = 0.43545 and RMSE = 0.50222, while [46] reported comparable prediction results of MSE = 0.53, MAPE = 7.6%, MAE = 0.53 and RMSE = 0. A further comparative analysis is performed with [47], which achieved results of MAE 1.146 and RMSE 1.613, while the third competitor SVR achieved an MAE of 1.367 and RMSE of 1.994 in [47]. However, MSE and MAPE were not considered in the evaluation of prediction models proposed in [47]. The comparative analysis proved the superiority of our proposed solar irradiance prediction model (CSA-ADAM optimized ANN model) by assigning CSA and ADAM optimized weights to the edges of the ANN.

**Table 2:** ‘Forecasting accuracy comparison of our proposed solar irradiance forecasting model.

Error Criteria	ANN [46]	ANN [47]	SVR [47]	ADAM-optimized ANN (Baseline)	CSA-ADAM optimized ANN (Proposed)
MSE	0.53	-	-	0.52420	0.252231
MAPE	7.6%	-	-	0.18197%	0.17055%
MAE	0.53	1.146	1.367	0.64649	0.43545
RMSE	0.62	1.613	1.994	0.72402	0.50222

### 6.2 Computational performance analysis

Using the same test dataset, we compared the prediction times of the ADAM-optimized ANN model (baseline) and our proposed CSA-ADAM optimized ANN model to determine their practical viability. To account for runtime variability, which is typical in shared environments, we timed the generation of predictions over a number of runs, that is, taken as 10 in this case.

According to the results, the CSA-ADAM optimized ANN model took 0.1110 ± 0.0058 seconds to make predictions on the test data, whereas the ADAM-optimized ANN model took an average of 0.1093 ± 0.0085 seconds. Both models show practical feasibility for near real-time forecasting tasks, although the hybrid model has a slightly longer prediction time because of its possibly more complex structure. The suggested approach provides increased accuracy with only a slight increase in computational cost.

### 6.3 Limitations and generalization

There are certain limitations to take into account, even though the suggested CSA-ADAM optimized ANN model exhibits encouraging gains in solar irradiance forecasting. The model’s applicability to other climates or locations without additional adaptation may be limited by the study’s reliance on a particular dataset from a specific geographic region and set of weather conditions. The model’s performance on data with varying temporal resolutions or in real-time forecasting scenarios has yet to be confirmed, as it was trained and tested on historical data gathered at predetermined 10-minute intervals per day for one week. Large-scale or real-time applications may also encounter difficulties due to the computational complexity brought about by the hybrid optimization, especially the CSA component.

In terms of generalizability, while the hybrid optimization framework successfully integrates local and global search, its efficacy is contingent upon the representativeness and quality of the features and input data. Other datasets may need different handling or additional feature engineering because the preprocessing steps and feature selection process used here were customized for this particular dataset. Furthermore, the model’s performance during various seasons and abnormal weather events was not thoroughly examined in the study, which may have affected forecast accuracy. In order to increase generalizability under a variety of circumstances, our future work may test the model on a variety of datasets, incorporate more reliable validation techniques like cross-validation, and look into adaptation techniques.

## 7 Conclusion and future work

Use of fossil fuels to generate electricity leads to higher energy cost and an increase in pollution. The demand for energy is increasing day by day. The answer is solar energy, which can be a solution to all rising electricity bills and environmental problems. Solar energy is a cheap and renewable source of energy. It is also a nature friendly and energy saving solution that is helpful in overcoming environmental impacts and reduce grid operating costs. However, the intermittent nature of solar energy has a negative impact on the smooth supply of electricity and grid stability. Therefore, a reliable and accurate forecasting model for solar energy is need of the hour. In this study, CSA-ADAM optimized ANN models is proposed to predict solar irradiance. Random weights assignment to teh edges of ANN is replaced with assignment of CSA optimized weights to the edges of ANN. Extensive simulation were performed to validate the prediction accuracy off our proposed CSA-ADAM optimized ANN forecasting model. Results of MSE = 0.252231, MAPE = 0.17055%, MAE = 0.43545, and RMSE = 0.50222 were achieved that prove supremacy of our proposed solar irradiance prediction model. In future, we plan to further improve the prediction accuracy of our proposed solar irradiance forecast model.

## Supporting information

S1 Dataset Solar irradiance dataset. Weather dataset used in this study. (ZIP)

## References

[1] Fang H, Akhayere E, Adebayo TS, Kavaz D, Ojekemi OR. The synergy of renewable energy consumption, technological innovation, and ecological quality: SDG policy proposals for developing country. Natural Resources Forum. 2024;49(1):561–77. doi: 10.1111/1477-8947.12404

[2] Khan S, Yuan H, Yahong W, Ahmad F. Environmental implications of technology-driven energy deficit and urbanization: insights from the environmental Kuznets and pollution hypothesis. Environmental Technology & Innovation. 2024;34:103554. doi: 10.1016/j.eti.2024.103554

[3] Gao PX, Curtis AR, Wong B, Keshav S. It’s not easy being green. SIGCOMM Comput Commun Rev. 2012;42(4):211–22. doi: 10.1145/2377677.2377719

[4] Mohsin SM, Maqsood T, Madani SA. Towards energy efficient cloud: a green and intelligent migration of traditional energy sources. Energies. 2024;17(11):2787. doi: 10.3390/en17112787

[5] Aslam S, Javaid N, Asif M, Iqbal U, Iqbal Z, Sarwar MA. A mixed integer linear programming based optimal home energy management scheme considering grid-connected microgrids. In: 2018 14th International Wireless Communications & Mobile Computing Conference (IWCMC). 2018. p. 993–8. 10.1109/iwcmc.2018.8450462

[6] Mohsin SM, Maqsood T, Madani SA. Solar and wind energy forecasting for green and intelligent migration of traditional energy sources. Sustainability. 2022;14(23):16317. doi: 10.3390/su142316317

[7] Akber SMA, Kazmi SN, Mohsin SM, Szczesna A. Deep learning-based motion style transfer tools, techniques and future challenges. Sensors (Basel). 2023;23(5):2597. doi: 10.3390/s23052597 36904801 PMC10007042

[8] Chomać-Pierzecka E, Kokiel A, Rogozińska-Mitrut J, Sobczak A, Soboń D, Stasiak J. Analysis and evaluation of the photovoltaic market in poland and the baltic states. Energies. 2022;15(2):669. doi: 10.3390/en15020669

[9] Aurangzeb K, Aslam S, Mohsin SM, Alhussein M. A fair pricing mechanism in smart grids for low energy consumption users. IEEE Access. 2021;9:22035–44. doi: 10.1109/access.2021.3056035

[10] Iqbal Z, Javaid N, Mohsin SM, Akber SMA, Afzal MK, Ishmanov F. Performance analysis of hybridization of heuristic techniques for residential load scheduling. Energies. 2018;11(10):2861. doi: 10.3390/en11102861

[11] Aurangzeb K, Aslam S, Haider SI, Mohsin SM, Islam S ul, Khattak HA, et al. Energy forecasting using multiheaded convolutional neural networks in efficient renewable energy resources equipped with energy storage system. Trans Emerging Tel Tech. 2019;33(2):e3837.doi: 10.1002/ett.3837

[12] Li W, Yang T, Delicato FC, Pires PF, Tari Z, Khan SU, et al. On enabling sustainable edge computing with renewable energy resources. IEEE Commun Mag. 2018;56(5):94–101. doi: 10.1109/mcom.2018.1700888

[13] Aslam S, Javaid N, Khan FA, Alamri A, Almogren A, Abdul W. Towards efficient energy management and power trading in a residential area via integrating a grid-connected microgrid. Sustainability. 2018;10(4):1245. doi: 10.3390/su10041245

[14] Agyekum EB, PraveenKumar S, Alwan NT, Velkin VI, Shcheklein SE. Effect of dual surface cooling of solar photovoltaic panel on the efficiency of the module: experimental investigation. Heliyon. 2021;7(9):e07920. doi: 10.1016/j.heliyon.2021.e07920 34522812 PMC8424511

[15] PraveenKumar S, Agyekum EB, Qasim MA, Alwan NT, Velkin VI, Shcheklein SE. Experimental assessment of thermoelectric cooling on the efficiency of PV module. Int J Renew Energy Res. 2022;12:1670–81.

[16] Asiaban S, Kayedpour N, Samani AE, Bozalakov D, De Kooning JDM, Crevecoeur G, et al. Wind and solar intermittency and the associated integration challenges: a comprehensive review including the status in the Belgian power system. Energies. 2021;14(9):2630. doi: 10.3390/en14092630

[17] Asiaban S, Kayedpour N, Samani AE, Bozalakov D, De Kooning JDM, Crevecoeur G, et al. Wind and solar intermittency and the associated integration challenges: a comprehensive review including the status in the Belgian power system. Energies. 2021;14(9):2630. doi: 10.3390/en14092630

[18] Dimd BD, Voller S, Cali U, Midtgard O-M. A review of machine learning-based photovoltaic output power forecasting: nordic context. IEEE Access. 2022;10:26404–25. doi: 10.1109/access.2022.3156942

[19] Li X, Ma L, Chen P, Xu H, Xing Q, Yan J, et al. Probabilistic solar irradiance forecasting based on XGBoost. Energy Reports. 2022;8:1087–95. doi: 10.1016/j.egyr.2022.02.251

[20] Ma Y, Lv Q, Zhang R, Zhang Y, Zhu H, Yin W. Short-term photovoltaic power forecasting method based on irradiance correction and error forecasting. Energy Reports. 2021;7:5495–509. doi: 10.1016/j.egyr.2021.08.167

[21] El Alani O, Abraim M, Ghennioui H, Ghennioui A, Ikenbi I, Dahr F-E. Short term solar irradiance forecasting using sky images based on a hybrid CNN–MLP model. Energy Reports. 2021;7:888–900. doi: 10.1016/j.egyr.2021.07.053

[22] Guo Z, Zhou K, Zhang C, Lu X, Chen W, Yang S. Residential electricity consumption behavior: influencing factors, related theories and intervention strategies. Renewable and Sustainable Energy Reviews. 2018;81:399–412. doi: 10.1016/j.rser.2017.07.046

[23] Aslam S, Herodotou H, Mohsin SM, Javaid N, Ashraf N, Aslam S. A survey on deep learning methods for power load and renewable energy forecasting in smart microgrids. Renewable and Sustainable Energy Reviews. 2021;144:110992. doi: 10.1016/j.rser.2021.110992

[24] Wang H, Lei Z, Zhang X, Zhou B, Peng J. A review of deep learning for renewable energy forecasting. Energy Conversion and Management. 2019;198:111799. doi: 10.1016/j.enconman.2019.111799

[25] Prema V, Bhaskar MS, Almakhles D, Gowtham N, Rao KU. Critical review of data, models and performance metrics for wind and solar power forecast. IEEE Access. 2022;10:667–88. doi: 10.1109/access.2021.3137419

[26] Huang X, Li Q, Tai Y, Chen Z, Zhang J, Shi J, et al. Hybrid deep neural model for hourly solar irradiance forecasting. Renewable Energy. 2021;171:1041–60. doi: 10.1016/j.renene.2021.02.161

[27] Kumari P, Toshniwal D. Extreme gradient boosting and deep neural network based ensemble learning approach to forecast hourly solar irradiance. Journal of Cleaner Production. 2021;279:123285. doi: 10.1016/j.jclepro.2020.123285

[28] Hendrikx NY, Barhmi K, Visser LR, de Bruin TA, Pó M, Salah AA, et al. All sky imaging-based short-term solar irradiance forecasting with long short-term memory networks. Solar Energy. 2024;272:112463. doi: 10.1016/j.solener.2024.112463

[29] Pereira S, Canhoto P, Salgado R. Development and assessment of artificial neural network models for direct normal solar irradiance forecasting using operational numerical weather prediction data. Energy and AI. 2024;15:100314. doi: 10.1016/j.egyai.2023.100314

[30] Yang M, Han C, Zhang W, Fang G, Jia Y. A short-term power prediction method based on numerical weather prediction correction and the fusion of adaptive spatiotemporal graph feature information for wind farm cluster. Expert Systems with Applications. 2025;274:126979. doi: 10.1016/j.eswa.2025.126979

[31] Wahidna A, Sookia N, Ramgolam YK. Performance evaluation of artificial neural network and hybrid artificial neural network based genetic algorithm models for global horizontal irradiance forecasting. Solar Energy Advances. 2024;4:100054. doi: 10.1016/j.seja.2024.100054

[32] Elmousaid R, Drioui N, Elgouri R, Agueny H, Adnani Y. Ultra-short-term global horizontal irradiance forecasting based on a novel and hybrid GRU-TCN model. Results in Engineering. 2024;23:102817. doi: 10.1016/j.rineng.2024.102817

[33] Liao Z, Coimbra CFM. Hybrid solar irradiance nowcasting and forecasting with the SCOPE method and convolutional neural networks. Renewable Energy. 2024;232:121055. doi: 10.1016/j.renene.2024.121055

[34] Gersnoviez A, Gámez-Granados JC, Cabrera-Fernández M, Santiago I, Cañete-Carmona E, Brox M. Neuro-fuzzy systems for daily solar irradiance classification and PV efficiency forecasting. Alexandria Engineering Journal. 2023;79:21–33. doi: 10.1016/j.aej.2023.07.072

[35] Subair A, G G. Solar irradiance forecasting using improved sample convolution and interactive learning. Procedia Computer Science. 2024;233:56–65. doi: 10.1016/j.procs.2024.03.195

[36] Etxegarai G, López A, Aginako N, Rodríguez F. An analysis of different deep learning neural networks for intra-hour solar irradiation forecasting to compute solar photovoltaic generators’ energy production. Energy for Sustainable Development. 2022;68:1–17. doi: 10.1016/j.esd.2022.02.002

[37] Faisal ANMF, Rahman A, Habib MTM, Siddique AH, Hasan M, Khan MM. Neural networks based multivariate time series forecasting of solar radiation using meteorological data of different cities of Bangladesh. Results in Engineering. 2022;13:100365. doi: 10.1016/j.rineng.2022.100365

[38] Assaf AM, Haron H, Abdull Hamed HN, Ghaleb FA, Qasem SN, Albarrak AM. A review on neural network based models for short term solar irradiance forecasting. Applied Sciences. 2023;13(14):8332. doi: 10.3390/app13148332

[39] Wentz VH, Maciel JN, Gimenez LJJ, Ando JOH. Solar irradiance forecasting to short-term PV power: accuracy comparison of ANN and LSTM models. Energies. 2022;15(7):2457.

[40] Sudharshan K, Naveen C, Vishnuram P, Krishna Rao Kasagani DVS, Nastasi B. Systematic review on impact of different irradiance forecasting techniques for solar energy prediction. Energies. 2022;15(17):6267. doi: 10.3390/en15176267

[41] Jacques Molu RJ, Tripathi B, Mbasso WF, Dzonde Naoussi SR, Bajaj M, Wira P, et al. Advancing short-term solar irradiance forecasting accuracy through a hybrid deep learning approach with Bayesian optimization. Results in Engineering. 2024;23:102461. doi: 10.1016/j.rineng.2024.102461

[42] Allal Z, Noura HN, Chahine K. Machine learning algorithms for solar irradiance prediction: a recent comparative study. e-Prime - Advances in Electrical Engineering, Electronics and Energy. 2024;7:100453. doi: 10.1016/j.prime.2024.100453

[43] Meng Q, He Y, Li S, Hussain S, Lu J, You G, et al. Adaptive two-step power prediction and improved perturbation method for accelerated MPPT with reduced oscillations in photovoltaic systems. Energy Reports. 2025;13:5328–38. doi: 10.1016/j.egyr.2025.04.055

[44] Sun J, Zhou S. Cuckoo search-ExtraTrees model for radio-frequency power amplifier under different temperatures. Frequenz. 2025;79(7–8):433–8. doi: 10.1515/freq-2024-0298

[45] Power data. https://power.larc.nasa.gov/data-access-viewer/

[46] Hamidreza J, Ahmadian A, Golkar MA, Elkamel A, Almansoori A. Solar irradiance forecasting based on the combination of radial basis function artificial neural network and genetic algorithm. In: Proceedings of the 6th European Conference on Renewable Energy Systems, Istanbul, Turkey, 2018. p. 25–7.

[47] Ghimire S, Deo RC, Downs NJ, Raj N. Global solar radiation prediction by ANN integrated with European Centre for medium range weather forecast fields in solar rich cities of Queensland Australia. Journal of Cleaner Production. 2019;216:288–310. doi: 10.1016/j.jclepro.2019.01.158

